# International medical graduates' social connections: A qualitative study

**DOI:** 10.1111/medu.15542

**Published:** 2024-09-30

**Authors:** Mo Al‐Haddad, Susan Jamieson, Evi Germeni

**Affiliations:** ^1^ College of Medical Veterinary and Life Sciences University of Glasgow UK; ^2^ Intensive Care Unit Queen Elizabeth University Hospital Glasgow UK; ^3^ School of Medicine Dentistry and Nursing College of Medical Veterinary and Life Sciences UK; ^4^ University of Glasgow, Health Economics and Health Technology Assessment (HEHTA) UK; ^5^ School of Health and Wellbeing, College of Medical Veterinary and Life Sciences University of Glasgow UK

## Abstract

**Introduction:**

Social connections in the host country improve International Medical Graduates' (IMGs') well‐being, intercultural competence and performance at work but is an issue that has been largely overlooked in the academic literature and policy discussions. The aim of this study was to better understand the social connections that IMGs form by exploring this phenomenon in a UK context.

**Methods:**

IMGs and UK Medical Graduates (UKMGs) practising in Scotland were invited to participate. This was a qualitative study using online semi‐structured interviews for data collection and reflexive thematic analysis.

**Results:**

Forty‐one participants were recruited (24 IMGs and 17 UKMGs), selected with maximum variation in terms of gender, ethnicity, speciality, grade and country of primary medical qualification. Twenty‐one (58%) of the participants had experience working in other parts of the UK.

Five themes were identified: (i) *overcoming early isolation*, IMGs strove to overcome their initial social isolation which harmed their mental well‐being; (ii) *where connections are made*, IMGs form social connections mainly at work and within their religious communities; (iii) *seeds of segregation*, some IMGs found themselves outside tight UK native friendship groups. Alcohol was a socially exclusive activity for some IMGs, as were other host country cultural norms. Exclusion led IMGs to form social connections with other IMGs or other ‘outsider’ groups; (iv) *degrees of Discrimination*, discrimination and racism were experienced by some IMGs. Discrimination was individual, structural and institutional, and (v) “*Open (ing) the door”*, participants described interventions at organisational, departmental, and individual levels to improve IMGs' ability to form social connections.

**Discussion:**

This study highlighted the challenges that IMGs face when trying to form social connections. More emphasis needs to be placed on promoting an environment where social connections, in particular between IMGs and host country natives, can flourish.

## Abstract 2

How do #IMGs form social connections? Why does it matter? Insights are provided through a large qualitative study conducted by @MoAlhaddad2, @SusanJHPE and Evi Germeni.

## 1 INTRODUCTION

A recent meta‐ethnography of qualitative studies highlighted commonalities in International Medical Graduates' (IMGs') experiences worldwide.^1^ For example, migration to a new country requires IMGs to overcome several challenges in order to thrive personally and professionally. These challenges – related to the dissonance between IMGs and the host country in terms of language, culture, medical education and belonging – contribute to the attainment gap between IMGs and Domestic Medical Graduates (DMGs),^1^, ^2^, ^3^, ^4^ and the higher risk of complaints made against IMGs.^5^, ^6^ The ability of IMGs to overcome these challenges is greatly enhanced by developing intercultural competence,^7^ which is also linked to better performance at work.^7^, ^8^, ^9^, ^10^ To develop intercultural competence, it is crucial for IMGs to establish social connections, especially with host country natives.^7^, ^10^, ^11^, ^12^ Social connections also mitigate some of the stress associated with migration and thus have a positive impact on migrants' well‐being.^13^, ^14^ However, the forming of social connections – especially outside the workplace – is an area that has received little attention so far in the literature on IMGs.^1^

**FIGURE 1:**
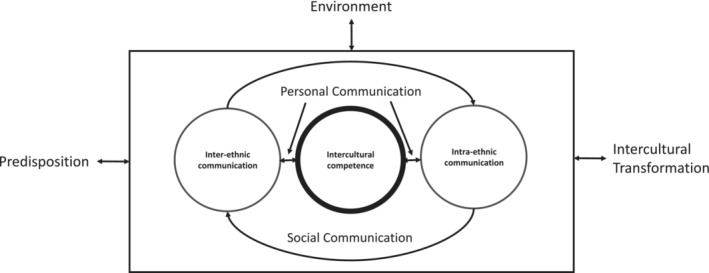
Factors affecting migrants' inter and intra‐ethnic communication and development of intercultural competence, from Kim's theory of cross‐cultural adaptation.^11^ Inter‐ethnic communication pertains to migrants' communication with members of other ethnic groups which in this context is the host country's dominant ethnic group. The ‘environment’ includes elements such as host receptivity. Figure modified with permission from Kim (2000).^11^

Other than the consumption of mass media in the host country, Kim posited that interactions with host country members in an individual or social context are the only way migrants can develop intercultural competence, Figure 1.^11^ Intercultural competence has been defined as the ‘*ability to communicate effectively and appropriately in intercultural situations based on one's intercultural knowledge, skills, and attitude*’.^15^, ^16^ However, migrants do not always have access to host country members to form these social connections. The reason is that this access depends on host receptivity, which is ‘*the natives’ openness toward strangers and willingness to accommodate strangers with opportunities to participate in local social communication processes'*.^11^ A highly receptive host provides ample interaction opportunities for migrants.^17^ However, host receptivity varies in the same host country depending on factors such as a migrant's cultural distance (which is the extent to which the migrant's culture differs from the dominant host country culture)^11^, ^18^ and skin colour.^19^

The literature focusing on the social connections of IMGs is scarce.^1^ Only a few empirical studies on this issue have been conducted,^20^, ^21^, ^22^ none of which included participants who were natives of the host country. Considering the equal role that host country natives play in forming these social connections,^12^ we sought to conduct an empirical study involving both IMGs and UK Medical Graduates (UKMGs)^23^ to better understand the social connections of IMGs in the UK. In addition to ease of access to participants for us, the UK is a particularly appropriate place to study this phenomenon as it has a long tradition of receiving IMGs (from a large number of countries) who now form 43% of the UK medical workforce.^24^, ^25^ The long tradition of receiving IMGs would make it likely that some of our older IMG participants could reflect on their experiences of social connections over time. Understanding IMGs' social connections in the UK would likely apply beyond this context considering the commonalities in IMGs' experiences worldwide.^1^ Our research questions were: (i) How do IMGs form their social connections? and (ii) How receptive a host is the UK perceived to be for IMGs?

## 2 METHODS

### 2.1 Theoretical framework

We approached our study from a constructivist worldview and drew from Kim's theory of cross‐cultural adaptation, Figure 1,^11^ when designing the interview guide (supplement 1). According to Kim,^11^ intercultural competence is developed by personal and social communication with host country natives (inter‐ethnic communication). This communication is affected by three factors: the migrant's predisposition, the environment (in the host country) and the intercultural transformation (the psychological and behavioural changes) of the migrant which, in turn, facilitates inter‐ethnic communication.

### 2.2 Participant recruitment

We recruited IMGs and UKMGs, who were working in Scotland, by advertising in a closed Facebook group of 791 members (Scottish IMG Doctors Support Network). In addition, we sent an email invitation to all doctors listed as trainees or trainers in the NHS Education Scotland database (6175 trainees and 5459 trainers) and to all Specialist and Associate Specialist doctors in Scotland (1225 doctors). We also advertised the study at a postgraduate educational event on diversity attended by 46 individuals.

Ninety‐three potential participants responded to our call, six from the Facebook post, one after the workshop announcement and the rest after our email campaigns. We selected the final participants using a purposive sampling technique with maximum variation^26^ in terms of gender, ethnicity, region of primary medical qualification (PMQ) and trainee/trainer status. MAH sent participants an information sheet, together with a consent form and a data privacy form which were signed and sent back by email. We achieved maximum variation and felt that we had sufficiently rich data after completing 41 interviews, which each took about one hour to complete. Although participants were recruited from Scotland, the majority had also worked in other parts of the UK, Table 1.

**TABLE 1:** Characteristics of participants.

	IMGs	UKMGs
Total number	24	17
Experience working outside Scotland in other parts of the UK	14	10
Trainers		
Consultants/GPs	12	12
SAS	1	
Trainees	11	5
Female*	13	8
Ethnicity*		
White**	8	13
Non‐white***	16	4
Country of PMQ		
Asia	7	
Middle east	5	
Africa	5	
Europe	6	
Other	1	17
UK		
Specialty		
Secondary care	20	15
Primary care	4	2

IMG = International Medical Graduate, UKMG = UK Medical Graduate, GP = General Practitioner (primary care physician), SAS = Specialist and Associate Specialist, Trainee = Doctor in training (e.g. residency programme) or similar level health board appointment, and PMQ = Primary Medical Qualification.

* *Self‐reported by participants. The rest of the participants self‐ reported as male*,

** *Terminology used for Scottish census 2022*,

*** *Grouped to preserve anonymity*.

### 2.3 Data collection

We collected data using online (Zoom or MS Teams) semi‐structured interviews (for the interview guide see supplement 1), which were recorded and transcribed using the software's auto‐transcription feature. MAH carried out all the interviews between June and November 2022. The interviews started by confirming participants' consent and willingness to participate, as well as asking participants to confirm their gender, ethnicity, country of PMQ, specialty and grade. After the interview, MAH corrected the auto‐generated transcripts and sent the corrected transcripts to participants to check for accuracy. To protect anonymity, we redacted identifying details.

### 2.4 Data analysis

All transcripts were imported into Nvivo 14 (QSR International, Doncaster, Australia). We analysed data inductively using the six‐phase reflexive thematic analysis approach described by Braun and Clarke.^27^ After an initial phase of data familiarisation, MAH carried out the initial data coding (phase 2). He also conducted the initial work of phase 3 (initial theme generation), phase 4 (theme development and review) and phase 5 (theme refining, defining and naming). He presented the initial work in each phase to SJ and EG at team meetings which took place about once a month. The work was discussed and challenged which led to further development and refinement of the themes. MAH wrote the initial draft of this manuscript, and all authors were involved in editing and revising it, phase 6 (writing a report).

### 2.5 Reflexivity

MAH (male) is an IMG of Iraqi origin who practices medicine in Scotland and has a national role in supporting IMGs. This study formed part of his PhD which employed qualitative research methods. MAH had his own beliefs and preconceptions in relation to the research questions before conducting the study. He developed these through personal experience, from reflection on his and others' experiences, and from the extensive review of the literature.^1^, ^7^ Honouring MAH's position and experiences was key in deciding to use the reflexive thematic analysis approach, where subjective storytelling by reflexively engaging with empirically collected data is valued.^27^, ^28^ As such, MAH's coding in phase one was inductive, but he was also sensitised to possible themes based on his experience and prior research.

MAH documented his preconceptions and thoughts in a reflexivity journal which he shared with his PhD supervisors SJ and EG. SJ (female) is a Scottish native and Professor (now Emeritus) of Health Professions Education. EG (female) is of Greek origin, has a social sciences background, and is a Reader in Qualitative Methods for Health Research. Both SJ and EG were involved in the study from conception through to data analysis and interpretation.

### 2.6 Ethics

We received ethical approval to conduct this study from a University of Glasgow ethics committee (Project No: 200210145). Considering MAH's seniority as a trainer in Scotland, we ensured that we minimised the potential power difference with participants by carefully wording the invitation to participate, emphasising that participation was voluntary. During interviews, MAH wore casual clothes, asked participants to address him by his first name and consciously used a relaxed and informal tone.

## 3 RESULTS

### 3.1 Participants

A total of 41 individuals took part in the study; 24 were IMGs and 17 UKMGs. All IMGs were born outside the UK and had migrated to the UK by choice; no one was a refugee. Participant characteristics are shown in Table 1; all participants had been in post for more than 6 months. Of 11 IMG trainees, seven were in their first post in the UK.

### 3.2 Themes and subthemes

We identified five main themes from the analysis of our data: *overcoming early isolation*, *where connections are made*, *seeds of segregation*, *degrees of discrimination* and “*open (ing) the door”*. We present these themes, sub‐themes and representative quotes in Table 2; and present further quotes in online supplement 2. In participant quotes, we have used squared brackets, [], to denote redactions and curved bracket, (), to provide explanation or context. We only report the ethnicity and gender of participants if that facilitates understanding of the context, e.g. Table 2, theme 4, subtheme 2.

**TABLE 2:** Themes, sub‐themes and representative quotes from participants.

Theme 1 – overcoming early isolation	
Sub‐theme	Quotes
Early isolation	IMG_Trainee08: “what was harder for me was at the end of the day when I had finished with work, everyone's going back to their own homes everyone had their own family, so I … I … didn't know really what to do, versus going back to an empty house and was just me.”
Characteristics enabling social connections	UKMG_Trainee02: “I think if you're a similar age to your colleagues, then that is naturally easier and that's what happens there … if you are a little bit older.”
The isolation‐avoidance‐isolation spiral	IMG_Trainee09: “And I, as I told you, I … I … after the first two months, I stopped going to the lunch breaks with the IMGs with the other Scottish and local doctors and other team. I stopped. I stopped the going there because I felt like …. I I I am bit isolated but when I did this, I felt more isolation.”
Being open and intentional	IMG_Trainee09: “As IMG I feel that no, you have to be more open to… to do the lunch breaks and to be in the coffee breaks, you know, and so that you will be. You will feel more part of the team rather than isolating yourself and it would be a vicious cycle of isolation.”
**Theme 2 – where connections are made**	
Sub‐theme	Quotes
Work, religion and other places	IMG_Trainer11: “Yeah, yeah, lots. Lots of of of friends who I I generally there are a few people whom I know both from work and from church.”
The importance of space and time	UKMG_Trainee05: “I stayed in the doctors' accommodations in [part of the UK]. They actually give you free accommodation for your F1 (first year post‐graduation) if you. If you want to live in doctors' accommodation. Not everyone does. So, I did. And because it's a couple, because it's [city in the UK] middle of nowhere. Everyone basically stays in the doctors' accommodation. And so that's probably the most socializing I've ever done with the group of doctors. And it was great. I loved every minute of it. Actually, we had a little doctors' mess and the accommodation with a TV and there wasn't really any kind of living spaces in the actual flats themselves. We just had a room in a small kitchen.”
Visiting homes	IMG_Trainer03: “We are quite a social group, and you know social ethnicity, we don't think twice about inviting someone over for dinner or for lunch or any any kind of social activity. But I haven't felt that kind of warmth coming from non [birth country] or non‐Asian; it's a cultural thing probably you know just invite people over.”
**Theme 3 – seeds of segregation**	
Sub‐theme	Quotes
Outside established friendship circles	IMG_Trainer07: “What is more difficult and other [from home country] have explained as well. So it's it's relatively easy to make contacts on the surface. OK to make … get people to know at first and come, but to make a deep friendship, to make a deep, you know, the people that … you into their inner core. I think there are people are relatively reserved here. There's just not that easy.”
Alcohol as an “exclusive social activity”	IMG_Trainee09: “For example, they when they are going to the to the, to the pub, for example they are. We are like having like some rounds while they are drinking and some people in and one each round, one pay for each round. But if some people just don't want to do this because you you don't want to pay for for the for the alcoholic drinks. You know what I mean? errm so you have to apologize for this.”
Connecting to other outsiders	IMG_Trainee04: “Scotland potentially is a bit, it seems like there's many more people who are very local …? To the bit more difficult to seems a bit more difficult because, like, I know they're from here, they started here, they are staying here. So firstly, they have like their I know partners and group of friends and stuff are pretty comfortable, not necessarily looking for new ones … err then maybe a bit less sort of open minded to people from other places as well, so a bit more trickier for them to be a bit more forward, maybe, errm … so yeah, so I got I got, friends with IMG as it usually ends up being I think.”
**Theme 4 – Degrees of discrimination**	
Sub‐theme	Quotes
Multidimensionality of discrimination	IMG_Trainee04: “yeah like people, people will respect a male doctor more than a female doctor … I think IMGs will be always a bit less … don't know reliable…or a bit more suspicious. That's my sort of personal feeling and sometimes even though I had very, very little … experience with someone being in like racist because of my nationality or something as well so maybe like a few small ahh small situations with like language or some like allusions or something but quite mild and nothing, nothing too big.”
Variable experience of discrimination	IMG_Trainer[redacted]: “I was in a bit in this … uh … uh … dream that Britain is so much better. You know, you just everyone's living happily together and and along each other. And then I went for a night out with a friend from Turkey, and that was a real eye opener because that was a Saturday night in in [town in Scotland] and and it was within the couple of hours, three times, you know, people were shouting at us from cars driving by or, you know, not necessarily in an totally aggressive way but but still … in a way I had not experienced this kind of ohh where do you? Where do you come, Feh (meaning from)?” White, IMG, man
**Theme 5 – “open (ing) the door”**	
Sub‐theme	Quotes
The onus is on the host	UKMG_Trainee01: “I think it's obviously; I don't think the onus should be on them to to integrate more, but I think they should be made to feel comfortable. And then I think if you're made to feel comfortable and welcome, then you do tend to integrate anyway … so it's it's a two‐way street.”
Organisational interventions	UKMG_Trainee02: “Get the the additional training that they (meaning IMGs) need either, and I also think that the the cultural aspect, I think, is often overlooked and it often not quite explained to them they're kind of left to sink or swim a little bit.”
Departmental interventions	IMG_Trainer01: “I think a consultants very simple thing is consultants having a meal with trainees is a very simple, informal way of and if consultants can, if their personal circumstances allow invite trainees to go out for her to a restaurant or to go out to invite them to their houses, not everyone's would that would be a very good, informal, welcoming way of, you know, making people feel at home.”
IMGs' approach	IMG_Trainer10: “Yeah, that's what I always tell tell my my trainees. Immerse yourself in the culture, immerse yourself, you know. Hey, just be part of it. I join, you know, never miss tea break. You know, don't lock yourself in the room. It is hard because like I experienced myself, it can be a very isolating experience …. Uh … particularly when you when you've just arrived.”

### 3.3 Overcoming early isolation

#### 3.3.1 Early isolation

IMGs described feeling isolated when they first arrived in the UK; “*I didn't have, really a social life*” (IMG_Trainee08), which was noticed by some UKMGs. The isolation lasted months to many years for some IMGs.

IMGs reported they just wanted “*to survive*” (IMG_Trainee01) at the start and did not have the energy to socialise, especially because they felt pressure to constantly present a different version of themselves. The isolation was exacerbated by exhaustion from communicating in English; “*I was knackered afterward purely because of using English all the time*” (IMG_Trainee08).

The language barrier – including that of the accent – led to a lack of confidence in socialising in English, which was also noted by UKMGs. IMGs were conscious that they might have come across as aloof or formal because of inadequate mastery of the language. They felt more comfortable speaking in front of other foreigners, as some perceived that the latter groups were more tolerant of their suboptimal mastery of English compared to natives. Others felt that natives were more tolerant. UKMGs commented that some IMGs stuck together, and it was difficult to get IMGs involved in social events inside and outside work; “*I don't know whether it's a language thing or (if) they just felt like we couldn't speak about the same things*” (UKMG_Trainee04). When IMGs had a good command of English, they recognised that it gave them an advantage when socialising. Being white was also acknowledged as an advantage in social settings.

Another barrier to socialising was unfamiliarity with cultural norms and references. Some IMGs were shy, afraid to make cultural faux pas or just didn't know how to contribute to conversations; “*I'm so careful now in social settings*” (IMG_Trainee11). Their lack of confidence in communicating was exacerbated in group settings; *“the difficulty (is) when you have like a group conversation”* (IMG_Trainee09).

Isolation, however, was not a universal experience, as some IMGs already had friends in the location where they landed, while others made friends quickly.

#### 3.3.2 Characteristics enabling social connections

In forming social connections, especially at work, participants felt that it helped if IMGs were female, young, single and at the same stage of life/training as their peers. There was an intersectionality in these factors, which meant that participants perceived older married men as the group who were more likely to be isolated; *“if you're a man with a family who's not living with you currently, … I think sometimes that's the hardest people to try and include”* (UKMG_Trainee02).

#### 3.3.3 The isolation‐avoidance‐isolation spiral

Socialising was perceived by IMGs as important for “*survival*” (IMG_trainer07) as it had a positive impact on their mental well‐being. Despite this, the difficulties in socialising and the lack of social networks led to anxiety and low self‐esteem for some IMGs. These in turn led them to further isolate themselves in a negative isolation‐avoidance‐isolation spiral; “*sometimes it's just easier to process your aloneness by yourself*” (IMG_Trainee11).

#### 3.3.4 Being open and intentional

Participants described that being open and intentional about socialising and forming friendships was key to succeeding. Sometimes IMGs had to force themselves to socialise; “*I think putting (in) that effort is necessary*” (IMG_Trainee06). There was recognition that openness and receptiveness must come from both IMGs and UKMGs; *“It does come from both”* (UKMG_Trainee04).

### 3.4 Where connections are made

#### 3.4.1 Work, religion and other places

There was a striking significance attached to the workplace and religious communities as a source of social connections for IMGs, especially soon after migration; “*my social circle is limited to the church and then people that I meet from work*” (IMG_Trainee02). IMGs spent a lot of time at their workplace and found that it provided a vital point of contact to develop social connections. On the other hand, UKMGs were deliberate in – and proud of – keeping their closer friendship groups separate from the work‐friends group; “*I think I've probably got more non‐medic friends*” (UKMG_Trainer06).

Some IMGs thought that it was easier to form social connections in areas with a high number of migrants; “*she (the IMG friend) moved down to [city in England] she said it was just a little easier where there's a lot more people who are like her*” (IMG_Trainer13), while others found it easier in smaller areas with fewer migrants. Clearly, that was influenced to some extent by the individual IMG's intention; “*I … didn't want to be only with [people from own country]*” (IMG_Trainer09). It was also influenced by how receptive the host environment was; “*I want to be as integrated as I will be allowed to be”* (IMG_Trainer09).

UKMGs were particularly sensitive to the role that socialisation played in improving mental health and bringing teams together; “*just being able to have lunch and sit down and have a chat with your colleagues actually makes a huge difference to your day*” (UKMG_Trainee05). However, communication at the workplace had certain rules that were understood by UKMGs but not necessarily by IMGs. One UKMG described it as a “*brand of communication*” (UKMG_Trainee04). When IMGs were not able to master that “*brand*”, they came across as bossy or rude; “*she was quite … dismissive and a bit rude*” (UKMG_Trainer06).

Some IMGs reached out to their religious communities early on and gained immediate access to thick^9^ and strong relationships; “*we joined the church as quickly as we could and that immediately opens up the social network*” (IMG_Trainer11). IMGs benefited from instrumental support from the religious communities to help them settle initially; “*they (the church) helped him (an IMG) find an area for the house*” (UKMG_Trainer04).

Other sources of social connections were joining group activities, classes or clubs that were related to sports – especially team sports – art, literature or learning a new language; “*so one of my colleagues introduced me to this [sport] group and I used to go play with them, even when she wasn't around, and we have social activities*” (IMG_Trainer03). In addition, some IMGs formed social connections through their children or with neighbours. Having a partner, family or a friend in the UK opened the door to other social networks.

#### 3.4.2 The importance of space and time

Both IMGs and UKMGs highlighted the importance of being with people in the same physical space for a long time to allow the forging of strong social connections. The hospital accommodation was described widely as a very important place for junior doctors to socialise and form strong social connections as was the doctors' mess in the hospital. Working in remote hospitals, especially if that hospital had accommodation, resulted in a positive social experience and making life‐long friends.

*“they had a really good mess culture at that time in England. So, we had a doctors' mess, and we still had these payday parties, all lived in hospital grounds, hospital accommodation … and hospital accommodation was actually really good.”* (IMG_Trainer08)

On the other hand, frequent rotations which led to changing teams meant that doctors were not always able to know their colleagues, and this led to disconnection, as did working as a locum (temporary) doctor or commuting long distances.

IMGs accepted that forming strong social connections needed time, with many describing an improvement in the quality and quantity of friendships they forged in the UK over the years, and a gradual increase in the proportion of UK natives within their social network; “*after years I can say that I have friends in Scotland*” (IMG_Trainer12).

#### 3.4.3 Visiting homes

IMGs noted that the culture of visiting homes and being invited to them was different in the UK. Inviting someone to your home to get to know them better was part of many IMGs' cultures, including European ones. However, UK natives (especially from a white background) would normally only invite people to their homes once they knew them well. This led to IMGs inviting UK natives to their homes, expecting the latter group to reciprocate, which often did not occur. IMGs interpreted this as British people being less “*warm”* (IMG_Trainer03) than people from the IMGs' birth countries or ethnic groups.

### 3.5 Seeds of segregation

#### 3.5.1 Outside established friendship circles

Sometimes IMGs found themselves in places that felt very ‘cliquey’ where social circles were already formed earlier in life, e.g. at school, during childhood, at university or early years of medical training. These social circles were very difficult to infiltrate, and IMGs were situated outside them; “*I do think that they (UK natives) are difficult to crack in terms of being invited to their social circle*” (IMG_Trainer06).

Apart from within religious communities, some IMGs commented that it was difficult to convert to friendships the connections that they made with colleagues at work or acquaintances from other places; “*I think it's harder here than it would have been for me back home*” (IMG_Trainee05). Some IMGs said that if they found friendships in the UK, these were not going to be as strong.

#### 3.5.2 Alcohol as an “exclusive social activity”

IMGs, including those from Europe, were struck by the central role of alcohol in most social activities in the UK. Consuming alcohol was recognised as an “*exclusive social activity*” (UKMG_trainer07) by both IMGs and some – not all – UKMGs. IMGs felt pressured at times to go to events where alcohol was consumed. Some IMGs ruled out attending work‐related social events because of alcohol consumption; “*I've never really sat inside of a pub… so, I have been invited on the group basis, but I just have never really gone*” (IMG_Trainee11). This aversion to heavy alcohol consumption was not limited to Muslims, but was also experienced by devout Christians, and some non‐religious IMGs.

#### 3.5.3 Connecting to other outsiders

IMGs described forming social connections more easily with other migrants or people who were not local, partly because they felt that members of the latter groups were “*people who underst (ood)*” (IMG_Trainee02). These social connections were formed with compatriots, people from the same region of origin, people of non‐white ethnicity, people of similar religion or anyone who was not local. The relationships with these ‘outsider’ groups were comforting, easy and formed either by choice soon after immigration or when IMGs faced rejection from UK natives; “*so I was very keen at first to have a lot of friendships from the English people in specific, but just ended up like diverting off that because they (the friendships) just couldn't work*” (IMG_Trainee03).

Forming social connections with ‘other’ people gave the ‘rejected’ IMGs comfort as they developed a sense of belonging to other outsider groups. Both IMGs and UKMGs recognised that exclusively bonding with other outsider groups hindered IMGs' social integration; “*you might end up not integrating*” (IMG_Trianer09).

### 3.6 Degrees of discrimination

#### 3.6.1 Multidimensionality of discrimination

Some IMGs experienced discrimination from managers, colleagues and members of the multidisciplinary team, patients or their relatives, or members of the public. IMGs' experiences of discrimination took many forms, including bullying, having unhelpful colleagues, mistrust, disrespect, hostility, lack of praise compared to UKMGs, stereotyping and public humiliation. There were also incidences of exclusion (e.g. from social events) or a more subtle lack of inclusion.

These experiences of discrimination were dismissed by some IMGs, as they felt that they were not sufficiently frequent; “*just twice … in my [6 month] placement, which is (a) very good number*” (IMG_Trainee01). For other IMGs, these experiences left a profound negative impact on their mental health and led some IMGs to leave their posts. IMGs felt that they had no‐one to go to when they experienced discrimination and feared talking about it in case it affected their career progression, which it sometimes did. Outright racism was experienced by some IMGs and had a devastating consequence.

*“I've walked down this street during the George Floyd trials (in the USA) because I was already here and I've had white young people walk up to me on the street and make that like a noose sign (makes a noose gesture) and actually said to me in one of the popular streets here in [city in Scotland] said to me ‐ I was walking with another black friend from [same country in Africa] ‐ and they had said the George Floyd … I CAN'T BREATHE.”* (IMG_Trainee11)

Allyship from UKMGs and IMGs helped mitigate some of the negative mental health effects of discrimination. However, allies did not always act at the time when discrimination happened, which left IMGs frustrated; “*I wish someone could have stood up for me then (quoting an IMG)*” (UKMG_Trainer12).

#### 3.6.2 Variable experience of discrimination

IMGs experienced discrimination because of accent, race, mastery of English, culture, religion, country of birth, name, gender (being female), or whether IMGs had a PhD, amongst other reasons. White IMGs experienced the least amount of discrimination and were acutely aware that their ‘whiteness’ protected them. Being a doctor was also seen as a protective factor against discrimination.

On the other hand, there were many examples of IMGs, some in senior leadership positions, who had not experienced discrimination after many years of living and working in the UK.

*“Maybe there is a glass ceiling of sorts; I have not really experienced it, my colleagues have been fantastic, and they've made me nothing but welcome”* IMG_Trainer09 (non‐white, Asian, man)

### 3.7 “Open (ing) the door”

#### 3.7.1 The onus is on the host

Participants suggested actions IMGs themselves could take but emphasised that the onus was on the host; “*when you put your hand out, it makes it easy for me to shake your hand*” (IMG_Trainee11); “*I think you need to open the door for somebody then to walk through it*” (UKMG_Trainee01).

#### 3.7.2 Organisational interventions

At the organisational level, the importance of space and time was re‐iterated by participants who advocated for re‐establishing hospital accommodation, doctors' messes and reducing the frequency of rotations. Cultural barriers could be broken down by introducing intercultural training within teaching programmes for colleagues, supervisors and other employees, providing cultural guidance to IMGs, or having a cultural buddy/sounding board. Diversity of cultures could be celebrated by multicultural events and providing a variety of foods in the workplace canteen. Another suggestion was appointing an IMG champion who would advocate for IMGs and to whom IMGs could go if they faced issues such as discrimination.

#### 3.7.3 Departmental interventions

The highest impact of measures to change the social environment was perceived to be those introduced at the department level. Measures could include an introduction to the department and key personnel in the department, and an orientation to the work culture in the department.

*“I think really the best place that it should come from … is from your colleagues, the people that you actually work with day‐to‐day.”* UKMG_Trainee05

Participants also mentioned simple things that colleagues could do to make IMGs feel welcome. For example, knowing IMGs as individuals, including them in social events that are culturally sensitive and simply showing them “*some hospitality*.” UKMG_Trainer11.

#### 3.7.4 IMGs' approach

Most participants suggested that there were things that IMGs themselves could do to improve social connections. For example, IMGs could be culturally open, curious and flexible when they come to the UK.

*“They've got to be open minded and try and understand the other culture and try and integrate as much as possible.”* IMG_Trainer04

At the same time, participants thought that IMGs need to be intentional in learning about the culture and language and developing intercultural competence. They could be intentional in seeking interpersonal interaction opportunities with a wide range of people, especially UK natives, by participating in social events, including joining colleagues during coffee and lunch breaks or even instigating the social events themselves. In addition, IMGs should strive to make their friendship groups more inclusive if possible.

Finally, participants recommended that IMGs should actively seek interpersonal interaction opportunities with UK natives and others by joining sports groups, art groups or getting involved in other local community activities.

## 4 DISCUSSION

### 4.1 Summary of principal findings

In answer to our first research question, we provided a rich picture of how IMGs formed social connections. We found that some IMGs felt isolated when they first arrived in the host country to the detriment of their mental well‐being. They overcame their isolation by finding social connections mainly at work and within religious communities. Some were successful in establishing social connections with host country natives, especially when both groups shared the same space for a long time. Other IMGs felt excluded or discriminated against and some experienced racism. Exclusion pushed IMGs to form social connections with other IMGs, members of other ‘outsider’ groups, or members of their religious communities. In answer to our second research question, we found that participants had mixed feelings about the UK's host receptivity. Some perceived the country as a hostile host, while others perceived it as a welcoming one.

### 4.2 Strengths and limitations

Our study relied on a large sample size of 41 participants and is one of the very few studies focusing on IMGs' social connections inside and outside the work environment. To our knowledge, it is also the only study on the topic which included host country natives. The inclusion of UKMGs in our study provided valuable insights that allowed us to investigate IMGs' social connections from different perspectives and enabled us to more fully understand the phenomenon as we will further discuss shortly.

Being an insider researcher facilitated conversations between the lead researcher and IMG participants. The seniority of the lead researcher might have encouraged some participants to express their views in the hope that he might influence change. Simultaneously, it might have prevented other participants from expressing their views openly.

Although our participants were recruited in Scotland, 24 (58%) had previously worked elsewhere in the UK, Table 1, and their views and experiences pertained more broadly to the UK context as demonstrated by some of the quotes. Overall, neither IMG nor UKMG participants noted major differences between Scotland and any other UK nation in terms of IMGs' social connections.

It was clear that UKMG participants were sympathetic to IMGs and that was what motivated them to participate in the study. Another potential weakness is therefore that we did not have UKMG participants who were less sympathetic.

Finally, none of our IMG participants were forced migrants and they were all settled in the UK. These issues are important as forced^29^ and temporary^30^ migrants are less likely to socially integrate.

### 4.3 Interpretation and relation to other studies

Our finding of early isolation is likely a risk for IMGs worldwide. It is an experience reported in the migration literature^13^, ^31^ and in studies on IMGs in other countries.^20^, ^21^ We found that this isolation had the potential to cause a profound negative impact on IMGs' mental well‐being. This aligns with a wealth of evidence demonstrating that social networks developed by migrants prevent the negative psychological outcomes associated with migration.^13^, ^32^, ^33^, ^34^, ^35^ Furthermore – and this becomes more important later,^7^ migrants who form social connections with host country natives and integrate tend to adjust better and have better psychological outcomes compared to those who are segregated.^36^, ^37^ The extent of isolation of our IMG participants and the impact on their mental well‐being was not universally appreciated by our UKMG participants, nor had IMG participants expressed these to UKMGs. At times UKMGs were unclear why IMGs often isolated themselves in social settings or only mixed with other IMGs. Furthermore, we identified some of the reasons behind the early isolation, e.g. exhaustion from speaking a second language, cultural unfamiliarity and uncertainty, inability to understand and contribute to topics that natives were discussing, and the difficulty in communicating in group settings. Again, these causes are likely to be universally experienced by IMGs worldwide. Appreciating the nature and causes of IMGs' early isolation would make it easier for host country natives to support their IMG colleagues by making adjustments in social settings. Practically, these adjustments could include involving IMGs in social discourse on topics they are likely to be familiar with, such as their experiences and journeys, or engaging in conversations with IMGs on a one‐to‐one basis or in small groups.

Including UKMG participants allowed us to better understand another social dynamic, which is the role of the workplace in the forming of social connections. For UKMGs, especially those who were geographically close to their earlier‐formed social networks, it was important to keep ‘friends’ separate from ‘work colleagues’. This contrasted with IMGs who were – by the act of migration – far away from their earlier‐formed social networks, a phenomenon common to migrants^31^ including IMGs worldwide.^1^, ^38^ For these IMGs, the workplace was centre‐stage for forming social connections. This finding has two main ramifications. The first is that host country natives (DMGs, colleagues, employers) who want to support their IMG colleagues should be cognisant of this. The second is that IMGs should have realistic expectations of forming social connections at the workplace and broaden their options of places to form them. Specifically, and as suggested by our participants, IMGs should seek to partake in recreational and social activities in the community at large^39^ with the aim of forming social connections, especially with host country natives.^18^

The seeds of segregation are sown early (early isolation) and have the potential to grow due to the broadly incongruous nature of some social behavioural patterns between IMGs, and UKMGs and other UK natives (e.g. visiting homes,^20^ and alcohol consumption^40^). Accepting that the details might be different in countries that are culturally distant from the UK, the incongruity itself (between social behavioural patterns of migrants and host country natives) is a universal phenomenon.^36^, ^41^, ^42^ Segregation, by definition is the reduced contact between migrants and host country natives.^36^ This reduction in interaction opportunities^17^ between migrants and host country natives, delays the acquisition of intercultural competence of migrants^11^, ^15^, ^16^ which is vital for migrants' work performance and economic success in the host country.^8^, ^9^, ^10^ Assuming the host country wants their IMGs to develop intercultural competence and perform well, they should aim to prevent segregation and encourage integration or assimilation.^38^ To this end, host country natives, including DMGs, should ‘open the door’ and ensure a hospitable environment for IMGs. Such an environment is more likely if host country natives themselves acquire intercultural competence,^12^ develop an awareness of the challenges that IMGs face when attempting to form social connections and support IMGs in overcoming these challenges, e.g. by providing ample interaction opportunities.^17^

Failure to form social connections with host country natives led some of our IMG participants to connect with ‘other outsiders’. This phenomenon was described in the rejection‐identification model proposed by Branscome et al,^43^, ^44^ and is similar to what Neiterman and Bourgeault observed in IMGs who migrated to Canada and formed social connections with other IMGs.^22^ On the other hand, some of our IMG participants sought social connection within their religious communities, an approach that has been shown to protect migrants against the negative psychological impacts of migration.^13^, ^45^ It is worth noting that, similar to the Swedish study on IMGs' friendships,^20^ some of our IMG participants failed to find ‘true friendships’, especially in the early stages. However, with ‘time’ and sharing the same ‘space’ with host country natives, many managed to find ‘true friendships.’ This highlights the importance of patience when IMGs seek these social connections and intentionally making ‘time’ to share that same ‘space’ with host country natives.

Other than host receptivity, the ‘environment’ factor in Kim's model, Figure 1, contains another relevant element which is host conformity pressure, ‘*the extent to which the (host) environment challenges strangers to adopt the normative patterns of the host culture and communication system*.’^11^ Kim does not make a distinction between the work environment and society at large. Indeed, some argue that the hospital environment is a ‘*microcosm of the larger society*.^46^ However, our findings suggest that there is a difference in conformity pressure between the two environments. IMGs felt host conformity pressure more acutely in the workplace. This resulted in them having to present a different version of themselves, *“many times you're just not yourself”* (IMG_Trainee04), to conform to the *“brand of communication”* (UKMG_Trainee04) expected. IMGs did not report this pressure in the larger society.

The ‘predisposition’ factor in Kim's model, Figure 1, contains three elements. The only modifiable one is ‘preparedness for change’.^11^ This element has been highlighted as important by other scholars in the migration and intercultural competence literature.^15^, ^16^ Our participants were conscious that ‘being open and intentional’ had helped them form social connections and recommended other IMGs adopt that approach as well. The ‘intercultural transformation’ factor, Figure 1, is likely to have played a role in the ability of IMGs to form strong social connections with time.

Finally, discrimination against IMGs worldwide has been extensively reported.^1^, ^42^, ^47^, ^48^, ^49^ Our study provides further empirical evidence that these incidences are ongoing, as harrowing accounts of racism, discrimination and exclusion were described in detail by some of our participants. In our study's context which is likely similar to other contexts, the absence of cultural orientation programmes^21^ together with the impatient expectation that IMGs conform to the host country's cultural norms,^1^, ^21^, ^42^, ^47^ and the lack of emphasis on developing intercultural competence of host country native colleagues and members of the multidisciplinary team, all suggest that this discrimination is systemic and institutional.^50^ Apart from humanitarian reasons, it is inconceivable that the education and training attainment gap between IMGs and DMGs^2^ will be eliminated if we do not put an end to discrimination and racism.^7^ Organisations and policymakers must take immediate action on these issues.

### 4.4 Implications

From the level of policymakers and healthcare organisations, down to individual DMGs and IMGs, we can all facilitate IMGs' forming of social connections. Organisations need to establish strategies to facilitate social connections to improve the intercultural competence of IMGs, DMGs and other employees in healthcare systems. Organisations and training programme directors should be cognisant of the importance of ‘space and time’. Comfortable spaces should be made available for doctors to spend time together,^51^ and rotations to different locations should be kept to a minimum. Whenever possible, employers should provide incentives for IMGs to partake in recreational and community activities that would allow them to develop social connections with natives of the host country. Departments and colleagues should also prioritise enabling IMGs to form social connections. These connections should be considered by all as a ‘must have’ rather than a ‘nice to have’. Educational supervisors, for example, should include social connections as a domain when reviewing the progress of IMGs.^52^ Colleagues of IMGs should ‘look out’ for IMGs, especially during the early stages of migration. Colleagues should also project genuine hospitality and be sensitive to the fact that IMGs are trying to re‐assemble their social networks. All involved should have open dialogues as to what are culturally acceptable social events and make sure that at least some organised social events are culturally acceptable to all team members. Finally, IMGs should approach the experience of migration with an open mind and be curious about the society and culture they migrate to. They should actively seek social connections, especially with host country natives, and view these connections as an integral part of their endeavours to develop intercultural competence which is vital for IMGs to thrive personally and professionally.

We also encourage further research on IMGs' social connections in other geographical and cultural contexts. Such research might bring to light further commonalities in experiences of IMGs' social connection and might also highlight context‐specific differences. We have demonstrated the importance of including DMGs in studies on social connections and encourage future researchers to include them as well. Finally, the difference in conformity pressure between the workplace and society at large is worth exploring further.

## 5 CONCLUSIONS

Social connections, especially with host country natives, are pivotal to develop IMGs' intercultural competence. However, there are many challenges that face IMGs when trying to form these social connections. These challenges could be overcome by ensuring policymakers, organisations, departments and colleagues instigate measures to improve host receptivity. IMGs should also be open to, and intentional in, forming social connections, especially with host country natives.

## AUTHOR CONTRIBUTIONS

**Mo Al‐Haddad:** Conceptualization; data curation; methodology; investigation; validation; formal analysis; visualization; project administration; writing – original draft; writing – review and editing. **Susan Jamieson:** Conceptualization; formal analysis; methodology; visualization; writing – review and editing; supervision. **Evi Germeni:** Supervision; conceptualization; methodology; visualization; writing – review and editing; formal analysis.

## Supporting information


**Data S1.** Supporting Information.

**Data S2.** Supporting Information.

## Data Availability

The data that support the findings of this study are available on request from the corresponding author. The data are not publicly available due to privacy or ethical restrictions.

## References

[1] Al‐Haddad M , Jamieson S , Germeni E . International medical graduates' experiences before and after migration: a meta‐ethnography of qualitative studies. Med Educ. 2022;56(5):504‐515. 34859484 10.1111/medu.14708

[2] General Medical Council . Tackling differential attainment. 2024.

[3] Federation of State Medical Boards (FSMB) . and National Board of Medical Examiners (NBME) . Performance Data|USMLE 2024.

[4] Medical Council of Canada . MCC annual report 2022–2023. Medical Council of Canada; 2023:53.

[5] General Medical Council . The state of medical education and practice in the UK 2015., London, UK, 2015.

[6] Elkin K , Spittal MJ , Studdert DM . Risks of complaints and adverse disciplinary findings against international medical graduates in Victoria and Western Australia. Med J Aust, Australia. 2012;197(8):448‐452. doi:10.5694/mja12.10632 23072241

[7] Al‐Haddad M . Facilitating international medical graduates' acculturation: from theory to practice. Med Educ. 2024;58(1):136‐148. 37524527 10.1111/medu.15175

[8] Harrison DA , Shaffer MA . Mapping the criterion space for expatriate success: task‐ and relationship‐based performance, effort and adaptation. Int J Hum Resour Manag. 2005;16(8):1454‐1474. doi:10.1080/09585190500220648

[9] Lancee B . The economic returns of immigrants' bonding and bridging social capital: the case of the Netherlands. Int Migr Rev. 2010;44(1):202‐226. doi:10.1111/j.1747-7379.2009.00803.x

[10] Terry DR , Quynh L . Social capital among migrating doctors: the “bridge” over troubled water. J Health Organ Manag. 2014;28(3):315‐326. doi:10.1108/JHOM-09-2012-0178 25080647

[11] Kim YY . Becoming intercultural: an integrative theory of communication and cross‐cultural adaptation. Sage Publications; 2000. doi:10.4135/9781452233253

[12] Al‐Haddad M , Lu P‐y . It takes two to tango: the ‘inter’ in intercultural competence. Med Educ. 2024;58(7):766‐768. 38290785 10.1111/medu.15319

[13] Bekteshi V , Kang S‐w . Contextualizing acculturative stress among Latino immigrants in the United States: a systematic review. Ethn Health. 2020;25(6):897‐914. doi:10.1080/13557858.2018.1469733 29792072

[14] Kamimura A , Samhouri MS , Myers K , et al. Physician migration: experience of international medical graduates in the USA. J Int Migr Integr. 2017;18(2):463‐481. doi:10.1007/s12134-016-0486-9

[15] Deardorff DK . Identification and assessment of intercultural competence as a student outcome of internationalization. J Stud Int Educ. 2006;10(3):241‐266. doi:10.1177/1028315306287002

[16] Ward C , Szabó Á . Affect, behavior, cognition, and development: Adding to the alphabet of acculturation. In: Matsumoto D , Hyisung CH , eds. The handbook of culture and psychology. 2nd ed. Oxford Academic; 2019:411‐446. doi:10.1093/oso/9780190679743.003.0020

[17] Kim YY . Toward an interactive theory of communication‐acculturation. Ann Int Commun Assoc. 1979;3(1):435‐453. doi:10.1080/23808985.1979.11923776

[18] Glass CR , Gómez E , Urzua A . Recreation, intercultural friendship, and international students' adaptation to college by region of origin. Int J Intercult Relat. 2014;42:104‐117. doi:10.1016/j.ijintrel.2014.05.007

[19] Vazquez LA , Garcia‐Vazquez E , Bauman SA , Sierra AS . Skin color, acculturation, and community interest among Mexican American students: a research note. Hisp J Behav Sci. 1997;19(3):377‐386. doi:10.1177/07399863970193009

[20] Povrzanović Frykman M , Mozetič K . The importance of friends: social life challenges for foreign physicians in southern Sweden. Community Work Fam. 2020;23(4):385‐400. doi:10.1080/13668803.2019.1599323

[21] Schumann M , Sepke M , Peters H . Doctors on the move 2: a qualitative study on the social integration of middle eastern physicians following their migration to Germany. Global Health. 2022;18(1):78. doi:10.1186/s12992-022-00871-z 36028861 PMC9412787

[22] Neiterman E , Bourgeault IL . Conceptualizing professional diaspora: international medical graduates in Canada. J Int Migr Integr. 2012;13:39‐57.

[23] Al‐Haddad M . International medical graduates: defining the term and using it consistently. BMJ Glob Health. 2024;9(8):e015678. doi:10.1136/bmjgh-2024-015678 PMC11331860 39153749

[24] General Medical Council . Key stats from the medical register. General Medical Council; 2024.

[25] Al‐Haddad M . European international medical graduates (IMGs): are we ignoring their needs and under‐representing the scale of IMG issues in the UK? J R Soc Med. 2024;117(2):52‐54. doi:10.1177/01410768241230804 38423124 PMC10949872

[26] Patton MQ . Qualitative research and evaluation methods. Sage Publications; 2002:4.

[27] Braun V , Clarke V . Thematic analysis: a practical guide. UK Sage; 2021.

[28] Gough B , Madill A . Subjectivity in psychological science: from problem to prospect. Psychol Methods. 2012;17(3):374‐384. doi:10.1037/a0029313 22799622

[29] David HP . Involuntary international migration: adaptation of refugees. Int Migr. 1969;7(3‐4):67‐105. doi:10.1111/j.1468-2435.1969.tb01062.x

[30] DaĞCioĞLu B , Artantaş A , Keskin A , Eray İ , Üstü Y , Uğurlu M . Social adaptation status of Syrian refugee physicians living in Turkey. Cent Eur J Public Health. 2020;28(2):149‐154. 32592561 10.21101/cejph.a5955

[31] Rogler LH . International migrations: a framework for directing research. Am Psychol. 1994;49(8):701‐708. doi:10.1037/0003-066X.49.8.701 8092613

[32] Ward C , Rana‐Deuba A . Acculturation and adaptation revisited. J Cross Cult Psychol. 1999;30(4):422‐442. doi:10.1177/0022022199030004003

[33] Rahman O , Rollock D . Acculturation, competence, and mental health among south Asian students in the United States. J Multicult Couns Dev. 2004;32(3):130‐142. doi:10.1002/j.2161-1912.2004.tb00366.x

[34] Ong ASJ , Ward C . The construction and validation of a social support measure for sojourners: the index of sojourner social support (ISSS) scale. J Cross Cult Psychol. 2005;36(6):637‐661. doi:10.1177/0022022105280508

[35] Yoo HC , Lee RM . Ethnic identity and approach‐type coping as moderators of the racial discrimination/well‐being relation in Asian Americans. J Couns Psychol. 2005;52(4):497‐506. doi:10.1037/0022-0167.52.4.497

[36] Berry JW . Acculturation: living successfully in two cultures. Int J Intercult Relat. 2005;29(6):697‐712. doi:10.1016/j.ijintrel.2005.07.013

[37] Ryder AG , Alden LE , Paulhus DL . Is acculturation unidimensional or bidimensional? A head‐to‐head comparison in the prediction of personality, self‐identity, and adjustment. J Pers Soc Psychol. 2000;79(1):49‐65. doi:10.1037/0022-3514.79.1.49 10909877

[38] Cohn S , Alenya J , Murray K , Bhugra D , De Guzman J , Schmidt U . Experiences and expectations of refugee doctors: qualitative study. Br J Psychiatry. 2006;189(1):74‐78. doi:10.1192/bjp.bp.1105.010975 16816309

[39] Kilpatrick S , Johns S , Vitartas P , Homisan M . Mobile skilled workers: making the most of an untapped rural community resource. J Rural Stud. 2011;27(2):181‐190. doi:10.1016/j.jrurstud.2011.01.003

[40] Byrne E , Brugha R , McGarvey A . 'A melting pot of cultures' ‐challenges in social adaptation and interactions amongst international medical students. BMC Med Educ. 2019;19(1):86. doi:10.1186/s12909-019-1514-1 30885174 PMC6423840

[41] Kim Y. Cross‐cultural adaptation. Oxford Research Encyclopedia of Communication; 2017. Accessed September 18, 2024. https://oxfordre.com/communication/view/10.1093/acrefore/9780190228613.001.0001/acrefore-9780190228613-e-21

[42] Alkhatib M , Hasan I , Ali A , Zaidi Z . Unveiling the invisible: challenges faced by Arab women international medical graduates in U.S. Academia. Acad Med. 2024. doi:10.1097/ACM.0000000000005822 39042416

[43] Branscombe NR , Schmitt MT , Harvey RD . Perceiving pervasive discrimination among African Americans: implications for group identification and well‐being. J Pers Soc Psychol. 1999;77(1):135‐149. doi:10.1037/0022-3514.77.1.135

[44] Schmitt MT , Spears R , Branscombe NR . Constructing a minority group identity out of shared rejection: the case of international students. Eur J Soc Psychol. 2003;33(1):1‐12. doi:10.1002/ejsp.131

[45] Adam Z , Ward C . Stress, religious coping and wellbeing in acculturating Muslims. J Muslim Mental Health. 2016;10(2). doi:10.3998/jmmh.10381607.0010.201

[46] Blumenfield S. The hospital center and aging: a challenge for the social worker. J Gerontol Soc Work. 1983; 5:35‐60.

[47] Klingler C , Marckmann G . Difficulties experienced by migrant physicians working in German hospitals: a qualitative interview study. Hum Resour Health. 2016;14(1):57. doi:10.1186/s12960-12016-10153-12964 27662831 PMC5034673

[48] General Medical Council . National Training Survey. General Medical Council; 2023.

[49] British Medical Association . Racism in medicine. BMA; 2022.

[50] Pincus FL . Discrimination comes in many forms: individual, institutional, and structural. Am Behav Sci. 1996;40(2):186‐194. doi:10.1177/0002764296040002009

[51] Health Education England . NHS staff and learners' mental wellbeing commission. Health Education England; 2019.

[52] Galema G , Duvivier R , Pols J , Jaarsma D , Wietasch G . Learning the ropes: strategies program directors use to facilitate organizational socialization of newcomer residents, a qualitative study. BMC Med Educ. 2022;22(1):247. doi:10.1186/s12909-022-03315-9 35382804 PMC8981951

