# Supplementary material for: International medical graduates' social connections: A qualitative study
Source: Med Educ. 2024 Sep 30;59(3):338–49. doi: 10.1111/medu.15542 (PMC11789848; doi:10.1111/medu.15542)
Supplement: Supplementary file 1 — Data S1. Supporting Information. [file MEDU-59-338-s001.docx]

# Supplement 1

## Interview schedule

### Checklist

- Confirm name
- Specialty
- Happy to record
- Make sure read the PIS, signed the forms
- Describe what the research process is and what I will do with the data
- Make sure happy

### IMGs

1. What made you decide to become a doctor?
2. Why did you decide to immigrate? And why Scotland?
3. Tell me about your journey from (…country) to becoming a doctor here in Scotland.
4. How did you find the work social environment when you first started working in Scotland?
   1. What were the challenges and opportunities?
   2. How did you find communication and forming friendships and relationships at work?
   3. Has your perception and experience changed over the years/months?
   4. What kind of things did you do with your friends? What kind of places to you meet up?
5. Were there any adjustments that you made since you came here? Tell me about these.
6. How welcoming did you find society and people in general when you first came here?
   1. How was it communicating with or forming relationships with people?
   2. Tell me about the personal and family relationships and friendships you have made since coming to Scotland
   3. What kind of things did you do with your friends? What kind of places to you meet up?
7. Is there an ethnic or religious community/group here in Scotland that you also feel part of?
   1. Tell me about you and your family’s interactions with members of this community
   2. What things/activities do you tend to do with members of this community? What language(s) do you speak?
   3. How do you think being part of this community affects the way you interact with people at work or in society at large? E.g., are you expected to behave in a certain way or do certain things?
8. Thank you for sharing your experiences and views. Can you please tell me if you have observed different encounters, experiences or views from other IMGs?
9. What is your perception of work/the NHS and societal attitudes in general towards IMGs?
10. Finally, do you have any thoughts or suggestions on how to make the work environment more welcoming for IMGs? And what IMGs can do to integrate?

### UKMGs

1. What made you decide to become a doctor?
2. Tell me about your experiences working as a doctor here in Scotland.
3. How do you think this compares with working elsewhere?
   1. Have you worked or seen any other health system?
4. How did you find the work environment when you first started working and subsequently?
   1. What were the challenges and opportunities?
   2. How did you find communication and forming friendships and relationships at work?
   3. Has your perception and experience changed over the years/months?
   4. What kind of things did you do with your friends? What kind of places to you meet up?
5. Tell me about your social network both inside and outside work? How did you form your personal and family friendships and what kind of things do you do?
6. Tell me about your experiences in interacting and communicating with IMGs.
   1. Tell me about positive and/or negative experiences you have had communicating and interacting with IMGs.
   2. What are the challenges and opportunities in communicating and interacting with IMGs?
   3. When you described your social network earlier, are any of your friends IMGs? How were those relationships formed? What kind of things do you do with them? What kind of places would you meet up with them?
7. What is your perception of work/the NHS and societal attitudes in general towards IMGs?
8. Finally, do you have any thoughts or suggestions on how to make the work environment more welcoming for IMGs? And what IMGs can do to integrate?
