# Supplementary material for: International medical graduates' social connections: A qualitative study
Source: Med Educ. 2024 Sep 30;59(3):338–49. doi: 10.1111/medu.15542 (PMC11789848; doi:10.1111/medu.15542)
Supplement: Supplementary file 2 — Data S2. Supporting Information. [file MEDU-59-338-s002.docx]

# Supplement 2

Table S1. Themes, sub-themes and further quotes.

| **Theme 1 - overcoming early isolation** | |
| --- | --- |
| Sub-theme | Quotes |
| Early isolation | IMG_Trainee02: “So I think, maybe things around you know errmm stuff maybe stuff on telly, stuff on you know people, maybe are talking about places they visited, or you know talking about stuff they do outside of work or, and that I just didn't understand.”  IMG_Trainee04: for a whole day, so I didn't, I didn't have… like power or will to chat with people at lunch, it was just literally my only chance to…to do nothing. And I guess that's your only, that's your, like at that time was that was your only opportunity to speak to people and socialise… and I was… this was just way too much for me…to which are sort of expected and I guess you're…sort of naturally being alienated because you don’t… you seem like you're not socialising but I’m… I was just too tired, cause I’m actually quite social I think overall”.  IMG_Trainee04 : “ I think that's probably quite tiring as well isn't it cause it's…many times you just you’re not yourself.”  IMG_Trainee04: “Just makes you not want to… socialise really on there in your free time that much especially, yeah I guess, especially with locals because with language I feel it's always… you will always get a bit more tolerance from…from foreigners than the locals. which always makes me laugh a bit, because you’d think they know the language better so sh...sh… should, so it should be easier for them to fill in the gaps, if you say something not right.”  IMG_Trainee04: “And, and I was sitting there in the small group of people, the other FYs and like I really I really struggled to even follow the conversation.”  IMG_Trainee07: “So communication was a bit difficult in the beginning because I'm not used to speak English all the time, so it was a bit difficult trying to translate everything I wanna say in English, and it was difficult for for me as well because of that the the Scottish accent.”  IMG_Trainer07: “I think when you came here as an FY one, well, you had no friends. You were almost you had to make friends to survive, and everyone else was in the same position. OK, so it was almost very easy to make friends.”  IMG_Trainer06: “I would see that the IMGs would all end up going for lunch together so they would kind of stick together versus the other trainees.”  IMG_Trainer10: “And then in the beginning, there was a definite language barrier because although I think my English was always quite reasonable and to be confronted with a all these various Scottish accents was a real challenge. And…and…and for probably two or three months I felt quite isolated.”  UKMG_Trainee04: “I thought it really really hard to to engage with the... But the [from Asian country] trainees that came over actually and because they… were… just tend to group together and then not not really socialise with you. I don't know whether it's a language thing or they just felt like we couldn't speak about the same things.”  UKMG_Trainer02: “…you know, people would say, oh, did you have a good weekend? And I'd say, well, I was on my own, and I went to the cinema on my own, and I went shopping on my own and…and then they go, oh, that's nice. But they wouldn't ask you out to spend any time with them. I mean, it took a while. And after two years there, yes, I did make some friends. But generally, there was just a sort of sense of we're all right. And you're an incomer.”  UKMG_Trainer05: “I think it was easy to see, probably in retrospect, how under-confident they were. I think much better one-on-one.” |
| Characteristics that facilitate social connections | IMG_Trainee10: “Uh forming friends when you are starting your 30s is very different from from friends in your 20s, it's… the relationships are mostly… mostly formal.”  IMG_Trainer08: “I enjoyed I wasn’t married then I wasn’t attached to anyone. I really enjoyed the sort of social life of a junior doctor. There were some things that I found challenging but people were very welcoming and that hospital in [city in UK] had lots of international medical graduates, people and wasn't just people from the [From home country], you know, there were people from Africa. There were people from the Middle East and from Libya, from Iraq. So…and there's lots of Europeans as well. So, working with German doctors, Spanish doctors. So, it was as cosmopolitan as. And I think that in some ways is really fortunate. I shared my flat with an [From home country] girl and a Spanish girl. And all those things helped. And I really enjoyed it.”  UKMG_Trainee01: “As as a male so to then, if you've got a another guy that your friendly with to then say or do you wanna do you want to go out for a pint or whatever? I think there's some things almost like a fear of a, maybe it is fear of rejection with maybe the way to to characterize it. And it's not, it's not cause it's an intimate sense and it's difficult sometimes to to then make friends. I think when you're when you are slightly older and because one you're concerned that the person you're trying to be friends with has already got established friends and doesn't want anymore friends errr they might be busy, they might have family commitments and things as well, which then does make it difficult as well and I think. It's one of those things that if you can't some day and they say or not cant this time, then do you then approach them again and say, do you want to go for pint then? Errm…I think I think it's maybe easier for females to do that with each other because it's much more like they're much more open to new friendships errm” |
| The isolation-avoidance-isolation spiral | IMG_Trainee08: “It made me feel..uum umm mean have made me feel a bit disappointed in myself that I wasn't really courageous enough to go out and speak with my colleagues a bit more…ummm and I’m a shy person in general, so…ahh…and it's very it's very easy to get to fall into that trap, where you just tend to avoid kind of social situations which make you a bit uncomfortable maybe and…uhhh…and the whole process of you being by yourself and it doesn't really it became the whole process becomes a bit anxiety provoking and…umm”  IMG_Trainee08: “When I moved to Scotland again it was kind of similar to working in [region in England] there was there were a lot of colleagues but yes everyone had their own family, and I went back to my own home…err. It was quite a bit of quite a bit…maybe a bit boring and a bit, but anxiety provoking with the Scotland was so far away from mainland England, I didn't really know a lot of people in Scotland, so it was pretty was pretty non-existent my social life I’d say, for the first one year…urrm”  IMG_Trainee09: “I was. I was feeling really stressed about it and I felt if the social part, if the social work with was more merciful…errr regarding the the whole stress, the whole…stress of the first job, I would say it was it would. It could be about a good…errr…It will be a good a better experience for me. But it wasn't like this was like a whole stressful situation that I have to get away from a…As as fast as I can.”  IMG_Trainer05: “It affects it affects everything... it changed my life, it changed my life and sometimes and I never…When…on occasion, I thought I have never felt such a failure…in such a failure as a person. Because because of that (meaning the way they felt excluded), in the career and as a failure, as in you know, like I said as a doctor.” |
| Being open and intentional | IMG_Trainee03: “I need to do some acculturization go listen to somebody talk about their ski trip, even though I absolutely have 0 interest in snow and 0 interest in skies.[rolls her eyes] whatever, I'm but if I'm expecting them to hear me out, which is why I'm going to try and go on Monday. And if the conversation starts and I'm the centre of that conversation and people are like, [IMG’s name] tell us about you, why should I have the problem if somebody talks about their ski vacation?”  UKMG_Trainer11: “I think I think there was, there was one chap that as a foundation doctor with and he was a [south east asia] graduate who came to the UK errmm but actually. How best to put this? ermm He he was very interested in in Scottish and English football and was outgoing in his nature and had an outstanding sense of humour. errm therefore he was an individual who would integrate very well, I think in almost any. Any setting, no matter where he was.” |
| **Theme 2 - where connections are made** | |
| Sub-theme | Quotes |
| Work, religion, and other places | IMG_Trainee06: “Obviously it's much easier to find friends through your studies or through your work, and at this age. So that's what happened.”  IMG_Trainer01: “So, I was very lucky that is as soon as my career in the NHS started I met [name of person] and he completely took me under his wing. I think he gave me a an amazing introduction to…uh Scotland.”  IMG_Trainer03: “It there was an attempt by people to meet up go for a drink. Have dinners together and that was very helpful I think they they went out of their way to have a night out when I started as a new consultant, just to you know have this as a welcoming event, where I get to know people.”  IMG_Trainer05: “And the consultants were very good, and I can tell you, the reason I think for the social interaction successful was because the interaction is, I think the from top down, is very good.”  IMG_Trainer06: “The nursing staff are super friendly. I've been invited out to drinks. I've been invited to people's houses.[laughs] You know, I've done more in the four months than I did in five years, so I found people to be very nice, very approachable.”  IMG_Trainer08: “The British kids, as they we were, we were all in our mid 20s. Then we're just very normal and didn't seem to have any expectations of me. And you know within the first couple of months I went to one of the SHO’s Hen do. I got invited to someone's house and yeah, so integration wasn't so difficult I think.”  IMG_Trainee08: “I definitely remember that for the first for the first six months when I saw my colleagues at my workplace like my FY2 FY1 colleagues, they told they asked me hey, we are going out for going to the canteen for lunch, do you want to come with me?”  MAH: “And, did you have any other friends they're outside work, did you manage to form new friendships and so on?”  Trainee08: “Not not really. Just probably just my friends from work”  IMG_Trainer08: “Yeah, I made, I made sort of two types of friends. One was the one, the one group where the [Hospital Specialty] registrars and SHOs that I was working with and some of them have, you know, continued to be lifelong friends. And the other group was because I had came with with child and my child started at the nursery. So, I was very eager to become friendly with the mothers at that nursery. And again, some of them have, you know, we've stayed friends now. So those were the two social groups. The neighbours were very lovely as well.”  IMG_Trainer10: “As I say the the choir we would go and then became kind of more closely related to to some of those guys and then you just go to the pub for a pint after your choir practice and then they do some actually found some running mates at school. So so started running with with one of them and then he he did his heel and then. And met up with another guy and I'm actually just back from playing tennis with him. Sounds very posh. I've never played tennis before and I am utterly rubbish, but it's great. And yeah, and then really the the usual stuff, we got really friendly with two or three couples who had kids at the same school and they organized things for the school together.”  IMG_Trainer13: “It is lonely being an IMG and I I I that's why the the one friend that we were gonna go swimming with she she moved down to [city in England] She said it was just a little easier where there's a lot more people who are like her.” |
| The importance of space and time | UKMG_Trainee05 I stayed in the doctor's accommodations in [part of the UK]. They actually give you free accommodation for your F1 if you. If you want to live in doctor's accommodation. Not everyone does. So, I did. And because it's a couple because it's [city in the UK] middle of nowhere. Everyone basically stays in the doctor's accommodation. And so that's probably the most socializing I’ve ever done with the group of doctors. And it was great. I loved every minute of it. Actually, we had a little doctor's mess and the accommodation with a TV and there wasn't really any kind of living spaces in the actual flats themselves. We just had a room in a small kitchen.” |
| Visiting homes | IMG_Trainee04 : “um so I guess… I guess, so I invited my flatmate to [birth country in Europe] like because I am having some time off after FY so I’m hoping to be home, and I say Oh, it would be so great, if you could come, and I can show you around it will be fun…but she never invited me back to her home…? which is sort of…it’s not that I expected it but yeah maybe I did expect it, I don’t know...it just, maybe not expected, but it's a bit weird she didn't.”  IMG_Trainee10: “I stay with them at their place. We speak Arabic. We do all kind of things [laughs] we cook together.”  IMG_Trainer08: “But imagine it's the same for many, many cultures. You do come together because you share food. The thing that struck me about the UK was people didn't ever I wasn't…I didn't have to be a big meal, but people were reluctant to invite you to their houses for food. So, I you know what, somebody knocked our door and just came to chat. I'd always say come in for a cup of tea. And if there was food, I would share the food. And I found that really interesting that others didn't feel the same way. I mean you just got accepted and I the friends that I've made have always been ones that have offered food right from the start. So, I do wonder whether eating together doesn't bring people together.”  IMG_Trainer08: “I was very lucky, I started the [hospital] and [name of friend] - It would if you ever came across him - and so [name of friend] was a registrar there and he just seemed very sort of intrigued by the fact that there was this girl that had turned up to work and said that she had a child and had no friends in [City in Scotland]. So, he did march me to his house that evening- and I had taken public transport because I wasn't sure about driving that day to work - and introduced me to his wife who wasn't a doctor and said, well, you've got to be [name]’s friend because she has no friends.” |
| **Theme 3 – unintentional exclusion** | |
| Sub-theme | Quotes |
| Being an outsider of tight friendship groups | IMG_Trainee10: “Mostly formal. It's like you are work-friends, but you're not friends, friends.”  UKMG_Trainee01: “Because of the friendship group I have it, it does mean that I like I've. I've got that core group as somebody that I I trust implicitly… errm that have been a large part of my life as well, which means that it's difficult for everyone else to break into that core group. And I think because everyone is is has grown up together, it would be difficult for people peripherally to almost…errm break into that core. I know that's not to say they wouldn't peripheral members and they wouldn't be closer relationships within Individuals within the group err but I think it would be difficult to break that core unless you had been friends for a number of years.” |
| Alcohol is an “exclusive social activity” | UKMG_Trainer07: “Both for the for the international medical graduates or the pregnant trainees, or the people from Europe, that it's not all about going to a pub. Because that's a very exclusive social activity.” |
| Connecting to other outsiders | IMG_Trainee08: “All the four of us we're all unmarried people we've just moved after we graduated from Medical School in our respective places in [another country in Asia] or [Country in Asia] and…And every time we meet up we maybe discuss work a bit and maybe discuss personal life, how it's going, and I feel a bit more in touch with them like similar to them, compared to maybe maybe some of my colleagues over here working working in Scotland.”  IMG_Trainer13: “I did feel a little bit a little bit out, but I I think that feeling was probably shared among the and the the the group of the the international medical graduates, you just didn't feel part of the…It's not I…I was never aware that I was actively excluded. But you didn't share their common history of of going to whatever, you know, the university, you didn't really know…How...do you know where to hang out or you just weren’t in the in Group? Always got the feeling that was a little bit little bit outside of that group and a lot of the the people that I was mostly friendly with were the, the, the international medical graduates.”  IMG_Trainer05: “Do you know what outside in the society with these people with whom I met only in [City in Scotland] you are less...you are less nasty nasty nasty is not the right word. Before..so before this before before before they say, for example, what when I first arrived when before I knew them [meaning the religious community], and the people will but horrible to you and you don't cope very well and you just lose hope…you…because you put so much value to being accepted by all these people who doesn't like you to start with, when I met these people [meaning the religious community] it doesn't really matter anymore, whether these people [UK natives] like me, don't like me, I really don't care. You know, society in general, people at work, I don't care you, like me, you don't like me doesn't matter. [Arabic phrase meaning done or finished] [waves hand in the air] that's how I see it.” |
| **Theme 4 – intentional exclusion** | |
| Sub-theme | Quotes |
| Discrimination | IMG_Trainee01: “they'll have like a big team with them in the ward round, so I approached, I’ve seen the patient like lying in bed with five to six doctors I haven't even seen or work with them before. And I started to ask the consultant, what he wants me to translate…what you want me to say for the patients, he said: aren't you a doctor? I said yes, I’m a junior doctor. Don't you know how to take history? I said Yes, I know how to take history, so ask her. I was shocked, I didn't know what he wants me to ask. Was there a specific question why and what you want me to do?”  IMG_Trainee01: “And when the junior doctors seen that things are not right from like from the consultant doing something for me which is doesn't seem right for them, the other junior doctors approach me telling me: this isn't right, this is shouldn't be happening, please speak to your educational supervisor, so I did that and my education supervisor was very supportive like hearing me doing everything he can do like to make me feel more supportive in work and just telling me what has been done is not Okay, and we will do our best, but this something like this doesn't happen again.”  IMG_Trainee11: “So I spent… when I was first new in hospital in a [specialty], especially in one branch of [specialty] I often struggled with when people were cold towards me. I wasn't sure what it was. Is it that they're treating you different because you look different? Are they treating you different because your clinical knowledge is sub-par? So, you're not sure what the coldness is about. or the third factor. Are they treating you different just because you're new and we don't know you people are always cautious around the unknown umm.”  IMG_Trainer05: “But that's I think the problem because that's how it started, because you don't talk back. You're too scared to talk back, one English is not very good, even though you are good in English, but when, you know, you're put in the confrontation suddenly yous yous you forgot how to speak English. You know it's not something natural to fight for your rights in English if English is not your first language. So yeah that's that's so and then two is because you're Asian and in Asia, we don't con, no, no confrontation, if people agro to you, you just smile hi okay like this and then three I’m a Muslim I don't do this this.[och och with hand gestures of fighting, laughs] is just I don't know if that's like not a Muslimy thing to do. You just like Okay, I accept it and that's it leave it, but they do it again and again and that's the problem and I, when I look back, that was a big problem. And I see that in, a one of the trainees that we have an Arab trainee from [country in middle east] who's very nice very kind that's the same thing. People will at him, you know at him, he's not that good, at him all the time he just smiled and I kinda go oh my God, this was I was like before.”  IMG_Trainer05: “I talked to the human resources because I am fair and I don't think this is good but that's the…it stopped it stopped for me and it stopped for other people other other….foreign people, not as bad, but the consequences or the the the sort of reprisals that you, you have to face as a person is this; you can never practice in that specialty again.”  IMG_Trainer12: “A comments from nursing staff about my accent or my. Uh, my surname? And all these things it's. It's funny, it's funny but but…for people that may be vulnerable can be quite traumatic.”  UKMG_Trainee01: “I think that that again doesn't just apply to IMGs, it's people of colour. It's also females as well. They get quite a lot of…errm and they get quite a lot of negative err reactions. Just even simple things and such as are you the nurse? And I think they when you combine several factors. So, if somebody is an IMG, a person of colour it and a female, you then get this…get this exaggerated response to absolutely everything. And and again, I think that's that is an element of society that haven't quite embraced what modern culture is errm…and I think it does. I can't Even…so…I would say that quite a lot of the people you would say are kind of within our kind of lower socioeconomic and educational backgrounds. However, I do also think you have your older middle-class population that are also deeply suspicious of IMGs and do treat them differently. errm because of that. And I think that again is…is a societal thing where your doctor errm when you were going to say something that's 60s, seventies, 80s, your doctor growing up was a white middle class male. And that's that's all it was. And even having a female doctor was revolutionary.”  UKMG_Trainee04: “If I can be not politically correct then. My observation and opinion is that the…the NHS has its way of working and again, it comes down to communication, training and what we produce out of UK medical schools and when someone comes along, and doesn't fit that mould or speaks with a slightly different accent then their competency is called into question because they've not gone into the same sort of training and it is only after the particular individual has proven themselves to be integrated or competent, despite the colour of their skin and their accent, then they are accepted and it's no longer a problem. But I do feel that that is something that does happen, whether consciously or subconsciously, in the NHS, and it happens across the board to doctors, nurses, allied health professionals, even to our porters and our cleaners. It just happens to all categories of stuff. And that's my that's my opinion of it and it's something I want to challenge and change, but I suppose I don’t really know how to start or go about it.”  UKMG_Trainer07: “I think when I say I think medicine inside [region in] Scotland is racist. I think it's about anyone who doesn't have a British accent. So, if you have a British accent, regardless of what colour you are, you do better than if you have any accent of any description.” |
| Degrees of discrimination | IMG_Trainee[redacted]: “I find people are very welcoming. There's. I never experienced any kind of nationalism or or racism is not not applicable to me. But no, I… xenophobia towards me.” White IMG man  IMG_Trainer[redacted]: “Uh, yeah, I must say I didn't have any problems. I found for me, but I'm. I'm obviously aware that I'm sort of white Caucasian. And you know from the external you cannot probably distinguish me very much from a Scottish person. So, there may be, but I didn't experience any. I think rather the opposite.”  IMG_Trainer[redacted]: “Let's put let let's put it this way. OK, so I don't know racism. I have not encountered myself in person, obviously because I'm a white Caucasian, perhaps not.” |
| **Theme 5 – making things better** | |
| Sub-theme | Quotes |
| The onus is on the host to be welcoming | IMG_Trainee11: “I think the the simple things are simple really. I think there're responsibilities on both sides.”  IMG_Trainer02: “You know, from both sides, both sides of the IMG coming into a workplace as new, as well as established people in in that workplace trying to help the IMGs coming into the department. It takes a conscious…it takes a conscious effort and time on both sides.”  IMG_Trainer08: “I I think the very fact that they have left their own country to come and work here, whether they've done it for you know, whatever their motives are, I don't think they, if they they've already jumped through all the hoops, they've written the PLAB exam, they've got a degree that's recognized with this country. I don't think they need to do anymore.” |
| What organisations can do | IMG_Trainee08: “If your seniors especially your supervisors a educational supervisors and clinical supervisors when when you're when you're starting a new job if they're a bit more aware that this is an IMG and Initially, when they moved to this country as a new doctor maybe their needs and their their immediate challenges and struggles are different to British graduates.”  IMG_Trainee09: “The others who are dealing with the IMG should have some induction on how to deal with IMGs. If we have, they have to know that we are coming from a different culture with different language, and these and the language would be a burden for us, especially at the beginning. they need to know this. I will say more merciful, more merciful with…with …with day-to-day conversation is they have to be like I don't have to, but they should like being like more accepting, more accepting on the.., and more or integrating, trying to engage others… other IMG in the in the…in the in their culture, you know.”  IMG_Trainer01: “You need to take these things on board, but I would love to run a course, but I don't know how politically correct it will be that you know simple things like learn about the history, learn about the culture, learn about how to present yourself, and there are books available. I mean, these are not things that I've invented. There is a book about etiquettes. There's a book about culture. There's a book about history. There's a book about British way of attire.”  IMG_Trainer02: “You know, from both sides, both sides of the IMG coming into a workplace as new, as well as established people in in that workplace trying to help the IMGs coming into the department. It takes a conscious…it takes a conscious effort and time on both sides.”  IMG_Trainer06: “A lot of the IMGs that I worked with. Their experience to medicine, to mine, to the UK, they're all so completely different and I think maybe even just delivering a talk on where they've come from, what they study journey was to make people more aware of the different paths that people take around the world to get to where they are now. I think a lot of people in the UK are ignorant for the amount of time, money, effort, studying that it takes to get to having that job in, in the system.  IMG_Trainer10: “And also as I said, you know we will not here to to change people's way to talk or to behave. But the recognition as as a as a work environment as a training environment that this is recognized that people are aware, OK this person might come across as rude, but that might not be because a person is rude but that is might be the way they they use English at the moment and they might need some help.”  IMG_Trainer13: “This is good because we didn't talk about race. It's it's very much a taboo thing and it wasn't until I actually asked my pal listen do you get lots of….That that I actually understood how difficult it must be, and I think we need to speak about it and maybe Scottish people need to… or not Scottish for the the the locals need to know how difficult it can be for doctors because a lot of the time the racist comments by patients for instance are are within a consultant consultancy atmosphere, where you are maybe one to one with the patient and nobody else hears it and I think…I think if if, if if people are aware how difficult it can be and how lonely it can be, it would be very useful.”  UKMG_Trainee01: “I do wonder actually whether, you know and and things like United States, you get foreign exchange students and things. I don't know what the what the classify it as now I'm just kind of on the bases it off errm off previous experience in TV. But I wonder whether actually there should be pathways, so this is this is much more of a a kind of school thing. So, people, it's not even just IMGs, but you get students coming from different cultures and different backgrounds errm and you also get the the ability to go to go the other way….errm would we integrate our kind of societal appreciation.”  UKMG_Trainee04: “Perhaps even for them, identifying a representative that they're comfortable approaching and speaking to that would then also be involved in the wider training group.”  UKMG_Trainer01 I think you know the other hospitals I worked there seemed to be a much greater select, you know, awareness of cultural differences in hospital. So, there will be different foods provided in the canteen. You know, I'm just, you know, and, again, it's in [hosp name] that seemed to be the best one. There was a variety, you know, even in the the shop there would be…that this was a variety. It was halal, there was Kosher, there was…and we worked with the Army medics who were obsessed about health. You know, there was something for everyone.”  UKMG_Trainer07: “So I think just having, I think making people aware that being an IMG is a very different experience from what they've (meaning UKMGs) been through and so, yeah…I mean, I think we, I think we need to start the conversation very early on.”  IMG_Trainer07: “The other thing I think what we're very useful and I'll be trying and this is something not is maybe for JHO or almost for like some sort of European, some sort of exchange program that you get trainee doctors, you know, maybe to work a year like for students you work a year in Italy you work a year in Spain you work a year and someone comes vice over over and this is something an idea I have and I haven't seen that anywhere but I'm sure if you would introduce this there probably would be…many people who would be interested in that and and that is something maybe I would like to see at some point.”  UKMG_Trainer10: “Actually celebrating the the numbers of languages that we use the number of faiths that that that there are…the number of disabilities, so that that the types of disabilities...that people have the joys and the sorrows that that comes with all of that. I think that's really good actually because if you understand the people, then it's much easier to to work together and celebrate the diversity rather than sometimes being a little bit nervous about asking questions.” |
| What departments and colleagues can do | IMG_Trainer03: “I think it'd be nice to have some built in situations like if you have a new training starting within the first two months, you must have you know some kind of a social gathering or every, at frequent interval there should be something going on.”  UKMG_Trainee02: “but then I also think that it is the onus is should be on.. people who are your UK based to try and have [inaudible] and include people because you're going to be nervous…like you've joined a new country… the culture’s like different…you could be on your own. And of course you're going to be nervous interacting with your colleagues outside of work because, like that's nervous and that's probably for the first time, especially nervous enough if you're not an IMG”  UKMG_Trainer07: “You know, maybe we need to be better at it, you know? You know, when I was a senior trainee in the [region in England], the consultants would host events, and we hosted a few events here actually for a few years.”  UKMG_Trainer07: “And are included in things and, and you know that we have we have very good trainee reps actually who we've just got new trainee reps who are very aware of EDI and they're making sure that their social events which they're incorporating are not all related about going to the pub, which obviously some people don't want to go to a pub quite right too. But you know, there's going to be gatherings in people’s houses, or they might go to a concert. Or so I think it's making sure. But IMGs need to be open about what they like and why. So, I suppose it's about them being honest about the activities they like.  UKMG_Trainer09: “I think the same things you would do with anyone to be welcoming is have a good induction, have a a mentor, have a supervisor, have a a errm I think have a coffee you know, and get to know people.”  UKMG_Trainer11: “I I think as a trainer, it's worth just trying as best as possible to get to know your trainees and being kind to them. And if you can show some hospitality.” |
| What IMGs can do | IMG_Trainee10: “It's OK. You shouldn't be saving money and not having a good life. Go, travel to Europe, do things. It's OK. I know this came comes from the survival mode, is that you want money to be safe but and here you made it. You can have fun.”  IMG_Trainer01: “I found that absence of curiosity, just mind numbing, absolutely mind numbing, they would in in when I was in the [Region in Scotland], I would often tell these people (meaning IMGs), you know, there's a there is a highland, you know, village festival being held. Go and see it. No, what will we do? We'll get bored. But you've never been there. How do you know you'll get bored.”  IMG_Trainer04: “They've got to be open minded and try and understand the other culture and try and integrate as much as possible. Because I have I have seen, you know, friends and colleagues who… even to this day will only socialize with people that go to their [religious building] or people that only speak their languages and have no white friends and it's just…it's almost like. they have failed to assimilate and they have…they are isolating themselves from the richness of what a different culture has got to offer.”  IMG_Trainer07: “Try to do something outside of medicine and and if you get into something outside of medicine, you then automatically meet other people and and forge contacts outside of medicine.”  IMG_Trainer09: “I think I think it works both ways it as an IMG coming in, you have to come in with an open mind.”  UKMG_Trainee04: “I suppose it there needs to be degree of openness, because I believe like you're coming to a different country, the culture is going to be different, and the working environment is gonna be different and you need to come with an expectation of that.”  UKMG_Trainer01: “I think it's just taking, you know, but it's like all things, sometimes you don't always feel like going on a work night out but you have to go.”  UKMG_Trainer01: “I think there has to be, you know, it's just being a bit more open.”  UKMG_Trainer01: “you need with all experiences you need a period of observing what is normal behaviour in that setting, you know, what are the patients like? The patients are different in every area that you go to and whether or not you're in a deprived area, less deprived or more country farm type aspect.” |
